# Socioeconomic differences in self-rated health among women: a comparison of St. Petersburg to Estonia and Finland

**DOI:** 10.1186/1475-9276-13-39

**Published:** 2014-05-17

**Authors:** Tatiana Dubikaytis, Tommi Härkänen, Elena Regushevskaya, Elina Hemminki, Elina Haavio-Mannila, Made Laanpere, Olga Kuznetsova, Seppo Koskinen

**Affiliations:** 1 St. Petersburg Medical Academy of Postgraduate Studies or North-Western State Medical University named after I.I. Mechnikov, 193015 Kirochnaja ul. 41, St. Petersburg, Russia; 2 The National Institute for Health and Welfare (THL), P.O. Box 30, Helsinki FI-00271, Finland; 3 Department of Social Research, University of Helsinki, Box 24, Helsinki 00014, Finland; 4 Department of Obstetrics and Gynaecology, University of Tartu, L. Puusepa 8, Tartu 50406, Estonia

**Keywords:** Health status, Education, Income, Health behaviour, Health disparities, Russia, Women

## Abstract

**Introduction:**

Social determinants of health have not been intensively studied in Russia, even though the health divide has been clearly demonstrated by an increased mortality rate among those with low education. A comparative analysis of social health determinants in countries with different historical and economic backgrounds may provide useful evidence for addressing health inequalities. We aimed to assess socioeconomic determinants of self-rated health in St. Petersburg as compared to Estonia and Finland.

**Methods:**

Data for women aged 18–44 were extracted from existing population-based surveys and analysed. In St. Petersburg the data were originally collected in 2003 (response rate 68%), in Estonia in 2004–2005 (54%), and in Finland in 2000–2001 (86%). The study samples comprised 865 women in St. Petersburg, 2141 in Estonia and 1897 in Finland.

**Results:**

Self-rated health was much poorer in St. Petersburg than in Estonia or Finland. High education was negatively associated with poor self-rated health in all the studied populations; it was (partially) mediated via health behaviour and limiting long-term illness only in Estonia and Finland, but not in St. Petersburg*.* High personal income and employment did not associate with poor self-rated health among St. Petersburg women, as it did in Estonia and Finland. In St. Petersburg housewives rather than employed women had better self-rated health, unlike the two other areas.

**Conclusion:**

Women’s self-rated health in St. Petersburg varied similarly by education but differently by income and employment as compared to Estonia and Finland. Education is likely the most meaningful dimension of women’s socioeconomic position in St. Petersburg. More research is needed to further clarify the pathways between socioeconomic position and health in Russia.

## Introduction

In many European countries growing awareness of health inequalities within and between countries has resulted in intensive research of the social health gradient. It has been shown that the influence of socioeconomic position on health is mediated by diverse mechanisms that operate on individual and societal levels [1-6]. The pattern of health inequality within countries can be shaped by national health promotion policy, social security regulations, and the economic and political environment. Therefore comparisons across countries of health inequality may yield a better understanding of the complex interrelationships between socio-economic position and health.

Self-rated health is a widely used measure for population health status. It correlates with physical health, functional capacity, psychological well-being, and is a significant predictor of morbidity, mortality and health care utilization [7-9]. Analysis of the correlations between socioeconomic position and self-rated health is often used to assess pathways in the development of health disparities.

Health inequalities in post-Soviet Russia are of particular interest. Economic and social changes in the 1990s along with an accumulation of social health hazards over a life-course resulted in a deterioration in population health, especially of those who had a less advantaged socioeconomic position [10,11]. Still, in Russia the social determinants of self-rated health have been studied mainly by international researchers and the data are limited. Studies have been conducted in different parts of the country (Kazan, Taganrog, Novosibirsk, Moscow and Pitkäranta) [12-17] and only a few have analysed the entire Russian population [10,18,19]. There are conflicting results on the socioeconomic variations in self-rated health: education was associated with better health in women and men in Taganrog [11] and only among women in Pitkäranta [11]; the association was substantial in Moscow men and weak among Moscow women [12]; household income correlated with better health in women, but not among men in Pitkäranta [11].

The contribution of health behaviour to socioeconomic self-rated health differences is unclear in Russia. An association between lifestyle-related risk factors and educational difference in cardiovascular mortality and morbidity, similar to that in western countries, has been found among Russian men, but among women the role of these factors was less obvious [20,21]. Therefore it is of interest to analyse lifestyle pathways between socioeconomic position and health among Russian women. Available statistics show that the overall level of women’s health as measured by deaths is much worse in Russia than in Finland [22]; Estonian women fall somewhere between [23].

The aim of the present study is to describe the socioeconomic self-rated health gradient and to assess the contribution of behavioural risk factors to this gradient among reproductive age women in St. Petersburg, Russia’s second biggest city, and to compare the results to two neighbouring countries: Estonia and Finland. Socioeconomic position was assessed using education, personal income and employment status. In addition we considered family structure and long-standing illness as potential confounders [24].

Estonia is another post-Soviet country and Finland is geographically very close to St. Petersburg. Earlier studies concerning self-rated health in Finland [25] and cross national comparisons [26,27] showed that self-perceived health was poorer in Estonia than in Finland. In both countries women with low education had poor self-rated health. In Estonia, but not in Finland economic inactivity was associated with poor self-rated health [27].

## Methods

In St. Petersburg an anonymous survey was conducted in 2003–2004 in two areas of the city [28,29]. The women were given the questionnaire to be completed during a health examination or at home and either collected or mailed later. A random sample of 2501 women aged 18–44 was drawn; 1719 women were contacted and 1152 (67%) participated in the survey. For a detailed description see earlier reports [28,29].

In Estonia, a postal survey was conducted in 2004–2005 [30]. A random sample (n = 5190) of all women aged 16–44 was drawn, anonymous questionnaires were posted (3472 in Estonian and 1718 in Russian language) and 2735 (54%) completed questionnaires were received.

In Finland the Health 2000 Health Examination Survey was conducted in 2000–2001 [31]. A two-stage cluster sample (N = 10 000) that was representative of the population aged 18 or older was drawn. The response rate was 80% for young adults (aged 18 to 29 years) and 89% for those aged 30 years or older [32]. The sample contained 2266 women aged 16–44, of which 86% participated. Most of the variables used in this study were collected in the interview at the respondent’s home. Information on income was obtained from a national register and BMI was based on measured weight and height.

The study questionnaires were prepared jointly by Finnish, Russian and Estonian researchers for the St. Petersburg and Estonian surveys with the aim of ensuring maximum comparability with the Finnish Health 2000 Survey, regardless of the different language structures. The questionnaires in St. Petersburg and Estonia were constructed at the same time and were similarly formulated using the questions from the Finnish survey as a model [28].

Our data analysis was restricted to females aged 18–44. We excluded pensioners (for illness or handicap, 3 in St. Petersburg, 22 in Estonia, 22 in Finland) and women with missing values in any of the studied variables. The proportion of women excluded was higher in St. Petersburg (24%) compared to Estonia (15%) and Finland (3%). The total numbers of women whose data were used in the analyses were 865 in St. Petersburg, 2141 in Estonia and 1897 in Finland.

### Variables

All variables with the exception of income data in Finland were based on self-reported information. The outcome measure was self-rated health, which was obtained with the question: “How satisfied are you with your health?” with a five-point response scale (1 – very satisfied; 2 – satisfied; 3 – neither satisfied nor dissatisfied; 4 – dissatisfied; and 5 – very dissatisfied). Self-reported limiting long-term illnesses were obtained with the question “Do you have any permanent or chronic illness or any defect, difficulty or injury that reduces your working capacity and functional ability: Yes/No? If yes, please specify the exact medical condition”. The reported limiting diseases were classified into four groups: mental, cardio-vascular, trauma & musculoskeletal diseases and other diseases, based on our awareness that those conditions could be differently distributed across socioeconomic groups.

Level of education was measured by the total number of study years completed. We used a categorisation that was applied previously in a comparative study using the same data [28]. In St Petersburg and Estonia education was categorised: <11, 11–13, 14–16 and >16 years of education; In Finland: ≤10, 11–12, 13–15, and ≤16 years. Personal income was classified into four quartiles; in Russia and Estonia income was self-reported, but in Finland it was obtained from a national tax authority. Family structure was classified based on a question about marital status and number of children under 18 years of age living with the respondent. Four categories were created: living with a partner (married or cohabiting) and a child/children; living only with partner; living without partner/children; living only with a child/children. Employment status was classified into four categories: employed, student, unemployed, or housewife, based on the following question: “What is your main economic activity: employed, unemployed, housewife, full-time student, retired, not employed or others?” Occupation was asked in each survey, but we could not categorise it in a comparable way in the three areas, and so occupation was not included in the analysis.

Behavioural factors used in the analysis included smoking and body mass index (BMI), which was calculated from weight and height. BMI indicates a misbalance between calorific consumption and spending, which can reflect a lack of physical activity and/or unhealthy diet. Smoking was defined based on the question “Have you ever smoked regularly at least one cigarette (cigar or pipe tobacco) daily for at least one year?” with further clarification which allowed for four groups: never, in past, occasional and current. We used self-reported height and weight in the Russian and Estonian survey while in Finland, height and weight were measured. BMI was classified into four categories: low weight (under 20), normal weight (20–24), overweight (25–29) and obesity (30 and over).

### Statistical analysis

Associations between the explanatory variables and self-rated health were analysed using ordinal logistic regression models, with estimated cumulative odds ratios (COR) presented. The outcome self-rated health was modelled as an ordinal outcome with five categories (1 as the best and 5 as the poorest), into cumulative odds ratios (COR). The COR represents the proportional change in the odds for a subject being in categories 1, …, k vs. in categories k + 1, …, 5 (for any 1 < =k < =4). If there were only two outcome categories, then the model would be the standard logistic regression model giving odds ratios. We did not dichotomize the outcome as information would have been lost as well as statistical power.

We have assessed the influence of the variables in the possible causal pathway using the elaboration analysis. If a variable of interest, for example education, has influence on the outcome, then the adjustment for intermediate variables such as the behavior risk factors or chronic diseases does not remove the association of education and the outcome. These intermediate variables have been added group-wise following the assumed causal pathway. First the socio-economic position, second the behavior risk factors and third the chronic diseases. In model I, adjustment for age was applied; in model II socioeconomic position factors were mutually adjusted; in model III smoking and BMI were additionally incorporated; and in model IV diseases were additionally included. The analysis was performed with STATA 11.0 (StataCorp, 2009). The age-adjusted distributions of the explanatory variables by country were estimated with logistic regression and the predictive margins method [33]. The country differences were tested with the Wald test. The age- adjusted distributions were based on the predictive margins method [33]. The significance of interactions between the country and the explanatory variables was tested with the Wald test to assess the possible effect-modification of the country.

## Results

Women’s background characteristics varied in the three areas (Table 1). St. Petersburg and Finnish women were most different from each other and Estonian women were in between. Mean income in Euros was notably higher in Finland than in Estonia and St. Petersburg. Smoking was more common in St. Petersburg, as were longstanding limiting illnesses.

**Table 1:** Age-adjusted means, proportions or distributions of the explanatory variables by country

	**p-value**^**c**^	**St. Petersburg**	**Estonia**	**Finland**
**Number of women**^**a**^		**865**	**2141**	**1897**
**Mean income per month (in euro)**	*p < 0.001*	130,1 ± 5,5	243,0 ± 4,3	874,9 ± 14,4
**Education, yrs**	*p < 0.001*			
< 11 (<10)^b^		5	6	6
11-13 (10–12)		41	45	27
14-16 (13–15)		42	35	38
17+ (16+)		11	13	30
**Family, %**	*p < 0.001*			
Partner & child/children		36	45	44
Only partner, no child/children		24	19	24
No partner no child/children		29	22	23
Only child/children, no partner		11	13	9
**Employment, %**	*p < 0.001*			
Employed		76	56	64
Student		11	24	16
Unemployed		3	4	9
Housewives		10	15	11
**BMI, %**	*p < 0.001*			
Under 20		26	26	16
20-24.9		49	51	52
25-29.9		16	17	22
30+		9	7	10
**Smoking, %**	*p < 0.001*			
Never		41	52	58
In past		14	18	14
Occasional		14	8	5
Current		31	22	23
**Chronic diseases, %**				
Yes vs. no	*p < 0.001*	38	24	29
**Mental diseases, %**	*p < 0.01*	1	1	2
**Trauma and musculoskeletal, %**	*p < 0.001*	6	7	9
**Cardio-vascular diseases, %**	*p < 0.001*	6	4	2
**Other diseases, %**	*p < 0.001*	25	13	16

^a^pensioners and women with missing values in any of the studied variables were excluded.

^b^years of education in brackets corresponds to Finnish population.

^c^Wald test for comparing countries.

Self-rated health varied notably by area (Table 2). The proportion of women who rated their health to be poor was notably higher in St. Petersburg than in Finland; women in Estonia were in between. The estimated CORs for the associations between the explanatory variables and self-rated health are presented in Tables 3, 4 and 5, for the St. Petersburg, Estonian and Finnish populations, respectively.

**Table 2:** Distribution of women by their age-adjusted self-rated health by country, %

	**St. Petersburg**	**Estonia**	**Finland**
(Number of women)	(865)	(2141)	(1897)
Very satisfied	6	16	56
Satisfied	30	48	36
Neither satisfied nor dissatisfied	32	23	6
Dissatisfied and very dissatisfied	32	12	2
Total	100	100	100

**Table 3:** **Cumulative OR with 95**% **CI for self-rated health (1 = best health and 5 = poorest health) by socioeconomic, behaviour factors and longstanding illness among women in St. Petersburg**

	**Model I age-adjusted**^**a**^	**Model II + SEP adjusted**^**b,c**^	**Model III + behavior risk factors**^**b,d**^	**Model IV + chronic diseases**^**b,e**^
**Education**	p^f^ < 0.01	p^f^ < 0.05	p^f^ <0.10	p^f^ ns
< 11 yrs	1.00	1.00	1.00	1.00
11–13 yrs	0.67 [0.41–1.09]	0.65^#^ [0.39–1.07]	0.64 [0.39–1.06]	0.65 [0.38–1.12]
14–16 yrs	0.51^**^ [0.32–0.82]	0.52^*^ [0.31–0.85]	0.54^*^ [0.33–0.89]	0.55^*^ [0.32–0.96]
17+ yrs	0.42^**^ [0.23–0.77]	0.40^**^ [0.22–0.75]	0.46^*^ [0.25–0.87]	0.46^*^ [0.23–0.91]
**Personal income**	p^f^ ns	p^f^ ns	p^f^ ns	p^f^ ns
Lowest quartile	1.00	1.00	1.00	1.00
2nd quartile	1.17 [0.80–1.69]	0.87 [0.56–1.36]	0.88 [0.56–1.38]	0.87 [0.55–1.39]
3rd quartile	1.00 [0.69–1.45]	0.77 [0.49–1.21]	0.82 [0.52–1.30]	0.79 [0.50–1.26]
Highest quartile	0.85 [0.60–1.21]	0.69 [0.44–1.08]	0.69 [0.44–1.08]	0.65^#^ [0.41–1.04]
**Employment**	p^f^ ns	p^f^ < 0.05	p^f^ < 0.10	p^f^ < 0.10
Employed	1.00	1.00	1.00	1.00
Student	0.77 [0.49–1.19]	0.74 [0.44–1.23]	0.78 [0.46–1.32]	0.66 [0.37–1.17]
Unemployed	1.32 [0.72–2.42]	1.04 [0.54–2.01]	1.04 [0.54–2.02]	1.09 [0.58–2.06]
Housewives	0.66^#^ [0.42–1.03]	0.47^*^ [0.27–0.83]	0.48^*^ [0.27–0.86]	0.50^*^ [0.28–0.89]
**Family structure**	p^f^ ns	p^f^ ns	p^f^ ns	p^f^ ns
Child & partner	1.00	1.00	1.00	1.00
Only partner	1.01 [0.74–1.38]	1.00 [0.72–1.38]	0.89 [0.64–1.25]	0.97 [0.68–1.38]
No children no partner	0.87 [0.62–1.22]	0.85 [0.59–1.23]	0.79 [0.55–1.14]	0.78 [0.54–1.13]
Only children	0.98 [0.67–1.44]	0.90 [0.61–1.34]	0.92 [0.62–1.37]	0.84 [0.55–1.28]
**Smoking**	p^f^ <0.10		p^f^ ns	p^f^ ns
Never	1.00		1.00	1.00
In past	1.26 [0.84–1.87]		1.19 [0.78–1.80]	1.23 [0.80–1.90]
Occasional	1.04 [0.74–1.47]		1.01 [0.71–1.44]	1.04 [0.72–1.50]
Current	1.44^*^ [1.07–1.93]		1.31^#^ [0.97–1.78]	1.34^#^ [0.98–1.82]
**BMI**	p^f^ <0.001		p^f^ <0.01	p^f^ <0.05
Under 20	1.24 [0.90–1.72]		1.27 [0.91–1.78]	1.16 [0.82–1.65]
20–24.9	1.00		1.00	1.00
25–29.9	1.66^**^ [1.19–2.30]		1.63^**^ [1.15–2.31]	1.45^*^ [1.02–2.06]
30+	2.44^**^ [1.57–3.79]		2.33^***^ [1.48–3.67]	1.95^**^ [1.23–3.12]
**Mental illness**	p^f^ ns			p^f^ ns
Yes vs. no	1.86 [0.15–22.4]			4.53 [0.26–77.66]
**Trauma & musculoskeletal**	p^f^ <0.001			p^f^ <0.001
Yes vs. no	2.31^***^ [1.44–3.70]			3.75 ^***^ [2.27–6.18]
**Cardiovascular illness**	p^f^ <0.001			p^f^ <0.001
Yes vs. no	2.79^***^ [1.86–4.19]			4.49^***^ [2.91–6.92]
**Other diseases**	p^f^ <0.001			p^f^ <0.001
Yes vs. no	3.15^***^ [2.40–4.14]			4.47^***^ [3.31–6.04]

SEP = socioeconomic position, BMI = body mass index.

^a^Model I SRH = age + one of the explanatory variables (education, personal income, employment status, family structure, BMI, smoking, chronic diseases).

^b^Elaboration analysis by adding a group of explanatory variables in the model allows one to assess the mediation effect of the group of new variables.

^c^Model II SRH = age + SEP variables (education + personal income + employment status + family structure).

^d^Model III SRH = age + SEP variables + behaviour variables (BMI + smoking).

^e^Model IV SRH = age + SEP variables + behaviour variables + chronic diseases.

^f^Wald test for significance of the cumulative OR difference between the categories of the variable, ^#^p < 0.1; *p < 0.05; **p < 0.01; ***p < 0.001; ns not significant.

**Table 4:** **Cumulative OR with 95**% **CI for self-rated health (1 = best health and 5 = poorest health) by socioeconomic, behaviour factors and longstanding illness among women in Estonia**^**a**^

	**Model I age-adjusted**^**b,c**^	**Model II + SEP adjusted**^**c,d**^	**Model III + behavior risk factors**^**c,e**^	**Model IV + chronic diseases**^**c,f**^
**Education**	p^g^ < 0.001	p^g^ <0.05	p^g^ ns	p^g^ ns
< 11 yrs	1.00	1.00	1.00	1.00
11–13	0.58^*^ [0.39–0.88]	0.62^*^ [0.41–0.94]	0.69^#^ [0.46–1.05]	0.77 [0.49–1.19]
14–16	0.45^***^ [0.29–0.68]	0.53^**^ [0.35–0.81]	0.62^*^ [0.41–0.96]	0.70 [0.44–1.11]
17+	0.38^***^ [0.24–0.61]	0.52^**^ [0.32–0.84]	0.65^#^ [0.40–1.08]	0.73 [0.43–1.22]
**Personal income**	p^g^ <0.001	p^g^ < 0.001	p^g^ <0.001	p^g^ < 0.01
Lowest quartile	1.00	1.00	1.00	1.00
2nd quartile	0.90 [0.71–1.15]	0.91 [0.71–1.16]	0.89 [0.69–1.14]	0.91 [0.70–1.18]
3rd quartile	0.69^**^ [0.53–0.90]	0.69^*^ [0.50–0.95]	0.68^*^ [0.49–0.93]	0.65^**^ [0.47–0.90]
Highest quartile	0.50^***^ [0.39–0.65]	0.53^***^ [0.39–0.73]	0.52^***^ [0.38–0.71]	0.58^**^ [0.42–0.79]
**Employment**	p^g^ <0.05	p^g^ ns	p^g^ ns	p^g^ ns
Employed	1.00	1.00	1.00	1.00
Student	1.04 [0.81–1.33]	0.89 [0.67–1.19]	0.91 [0.69–1.21]	0.82 [0.61–1.10]
Unemployed	1.84^**^ [1.22–2.78]	1.18 [0.76–1.82]	1.16 [0.74–1.80]	1.31 [0.83–2.07]
Housewives	1.07 [0.82–1.38]	0.84 [0.62–1.14]	0.83 [0.62–1.13]	0.78 [0.58–1.06]
**Family structure**	p^g^ <0.05	p^g^ <0.05	p^g^ <0.10	p^g^ ns
Child & partner	1.00	1.00	1.00	1.00
Only partner	1.02 [0.81–1.30]	1.10 [0.86–1.41]	1.10 [0.86–1.41]	1.00 [0.78–1.28]
No children no partner	1.00 [0.78–1.27]	1.02 [0.78–1.33]	1.06 [0.81–1.38]	1.06 [0.81–1.40]
Only children	1.47^**^ [1.13–1.90]	1.46^**^ [1.12–1.90]	1.45^**^ [1.11–1.89]	1.35^*^ [1.02–1.78]
**Smoking**	p^g^ <0.001		p^g^ <0.001	p^g^ < 0.01
Never	1.00		1.00	1.00
In past	1.27^*^ [1.01–1.58]		1.29^*^ [1.03–1.62]	1.22^#^ [0.97–1.54]
Occasional	1.32^#^ [0.9–1.81]		1.25 [0.91–1.72]	1.21 [0.88–1.66]
Current	1.75^***^ [1.41–2.17]		1.58^***^ [1.26–1.98]	1.51^***^ [1.20–1.90]
**BMI**	p^g^ <0.001		p^g^ < 0.01	p^g^ <0.05
Under 20	0.97 [0.79–1.19]		0.96 [0.78–1.18]	0.92 [0.75–1.13]
20–24.9	1.00		1.00	1.00
25–29.9	1.38^*^ [1.08–1.76]		1.33^*^ [1.04–1.69]	1.36^*^ [1.07–1.75]
30+	1.90^***^ [1.34–2.71]		1.69^**^ [1.18–2.43]	1.42^#^ [0.96–2.09]
**Mental illness**	p^g^ <0.001			p^g^ <0.001
Yes vs. no	4.48^***^ [2.38–8.41]			7.55^***^ [3.61–15.78]
**Trauma & musculoskeletal illness**	p^g^ <0.001			p^g^ <0.001
Yes vs. no	3.64^***^ [2.67–4.98]			4.90^***^ [3.46–6.94]
**Cardiovascular illness**	p^g^ <0.001			p^g^ <0.001
Yes vs. no	4.02^***^ [2.73–5.93]			4.86^***^ [3.21–7.38]
**Other diseases**	p^g^ <0.001			p^g^ <0.001
Yes vs. no	4.09^***^ [3.24–5.16]			5.53^***^ [4.28–7.16]
**Language** Russian vs. Estonian	p^g^ < 0.001	p^g^ < 0.001	p^g^ < 0.001	p^g^ <0.001
	2.51^***^ [2.05–3.07]	2.12^***^ [1.72–2.61]	2.05 [1.66–2.53]	1.92 [1.55–2.38]

SEP = socioeconomic position; BMI = body mass index.

^a^All the associations were adjusted for language Russian vs. Estonian.

^b^Model I SRH = age + one of the explanatory variables (education, personal income, employment status, family structure, BMI, smoking, chronic diseases).

^c^Elaboration analysis by adding a group of explanatory variables in the model allows one to assess the mediation effect of the group of new variables.

^d^Model II SRH = age + education + personal income + employment status + family structure (SEP variables).

^e^Model III SRH = age + SEP variables + BMI + smoking.

^f^Model IV SRH = age + SEP variables + behaviour variables + chronic diseases.

^g^Wald test for significance of the cumulative OR difference between the categories of the variable.

^#^p < 0.1; *p < 0.05; **p < 0.01; ***p < 0.001; ns not significant.

**Table 5:** **Cumulative OR with 95**% **CI for self-rated health (1 = best health and 5 = poorest health) by socioeconomic, behaviour factors and longstanding illness among women in Finland**

	**Model I age-adjusted**^**a**^	**Model II + SEP adjusted**^**b,c**^	**Model III + behavior risk factors**^**b,d**^	**Model IV + chronic diseases**^**b,e**^
**Education**	p^f^ < 0.001	p^f^ <0.001	p^f^ <0.001	p^f^ <0.001
<10 yrs	1.00	1.00	1.00	1.00
10–12 yrs	0.69^#^ [0.46–1.02]	0.78 [0.52–1.16]	0.83 [0.56–1.24]	0.87 [0.58–1.30]
13–15 yrs	0.52^**^ [0.36–0.76]	0.60^**^ [0.41–0.88]	0.69^#^ [0.47–1.01]	0.77 [0.52–1.14]
16+ yrs	0.35^***^ [0.23–0.52]	0.40^***^ [0.27–0.60]	0.47^***^ [0.31–0.72]	0.51^**^ [0.34–0.77]
**Personal income**	p^f^ <0.001	p^f^ <0.01	p^f^ <0.01	p^f^ <0.01
Lowest quartile	1.00	1.00	1.00	1.00
2nd quartile	0.61^**^ [0.46–0.81]	0.68^**^ [0.51–0.89]	0.71^*^ [0.53–0.94]	0.75^#^ [0.56–1.00]
3rd quartile	0.47^***^ [0.35–0.62]	0.58^**^ [0.43–0.79]	0.60^**^ [0.44–0.82]	0.63^**^ [0.46–0.86]
Highest quartile	0.51^***^ [0.38–0.68]	0.74^#^ [0.53–1.02]	0.79 [0.57–1.10]	0.85 [0.61–1.17]
**Employment**	p^f^ <0.001	p^f^ ns	p^f^ ns	p^f^ ns
Employed	1.00	1.00	1.00	1.00
Student	1.31^#^ [0.96–1.78]	1.19 [0.85–1.67]	1.26 [0.90–1.77]	1.20 [0.86–1.69]
Unemployed	2.10^***^ [1.50–2.92]	1.58^*^ [1.08–2.31]	1.53^*^ [1.05–2.24]	1.31 [0.90–1.90]
Housewives	1.40^*^ [1.01–1.92]	1.21 [0.86–1.71]	1.24 [0.88–1.75]	1.13 [0.78–1.63]
**Family structure**	p^f^ <0.10	p^f^ ns	p^f^ ns	p^f^ <0.05
Child & partner	1.00	1.00	1.00	1.00
Only partner	1.04 [0.81–1.34]	1.12 [0.85–1.47]	1.13 [0.86–1.48]	1.17 [0.89–1.53]
No children no partner	0.95 [0.71–1.26]	0.91 [0.67–1.24]	0.84 [0.62–1.15]	0.80 [0.58–1.10]
Only children	1.50^*^ [1.08–2.09]	1.24 [0.89–1.73]	1.26 [0.89–1.76]	1.29 [0.90–1.84]
**Smoking**	p^f^ <0.001		p^f^ <0.01	p^f^ <0.001
Never	1.00		1.00	1.00
In past	1.05 [0.81–1.36]		0.92 [0.70–1.21]	0.95 [0.72–1.25]
Occasional	0.61^*^ [0.38–0.99]		0.56^*^ [0.34–0.92]	0.50^**^ [0.30–0.84]
Current	1.60^***^ [1.29–1.98]		1.29^*^ [1.02–1.62]	1.32^*^ [1.04–1.67]
**BMI**	p^f^ <0.001		p^f^ <0.001	p^f^ <0.001
Under 20	1.27^#^ [0.97–1.67]		1.19 [0.90–1.57]	1.21 [0.92–1.58]
20–24.9	1.00		1.00	1.00
25–29.9	1.40^**^ [1.13–1.75]		1.34^*^ [1.07–1.68]	1.34^*^ [1.06–1.70]
30+	3.02^***^ [2.28–4.02]		2.85^***^ [2.13–3.81]	2.36^***^ [1.75–3.18]
**Mental illness**	p^f^ <0.001			p^f^ <0.001
Yes vs. no	10.4^***^ [5.86–19.15]			10.90^***^ [5.41–21.96]
**Trauma & musculoskeletal**	p^f^ <0.001			p^f^ <0.001
Yes vs. no	4.36^***^ [3.26–5.83]			4.57^***^ [3.34–6.25]
**Cardiovascular illness**	p^f^ <0.001			p^f^ <0.001
Yes vs. no	3.76^***^ [2.17–6.52]			3.02^***^ [1.72–5.31]
**Other diseases**	p^f^ <0.001			p^f^ <0.001
Yes vs. no	1.82^***^ [1.44–2.30]			2.59^***^ [1.98–3.39]

^a^Model I SRH = age + one of the explanatory variables (education, personal income, employment status, family structure, BMI, smoking, chronic diseases).

^b^Elaboration analysis by adding a group of explanatory variables in the model allows one to assess the mediation effect of the group of new variables.

^c^Model II SRH = age + education + personal income + employment status + family structure (SEP variables).

^d^Model III SRH = age + SEP variables + BMI + smoking.

^e^Model IV SRH = age + SEP variables + behaviour variables + chronic diseases.

^f^Wald test for significance of the cumulative OR difference between the categories of the variable.

^#^p < 0.1; *p < 0.05; **p < 0.01; ***p < 0.001; ns not significant.

SEP = socioeconomic position.

### Variation in self-rated health by socioeconomic position

**Figure 1:**
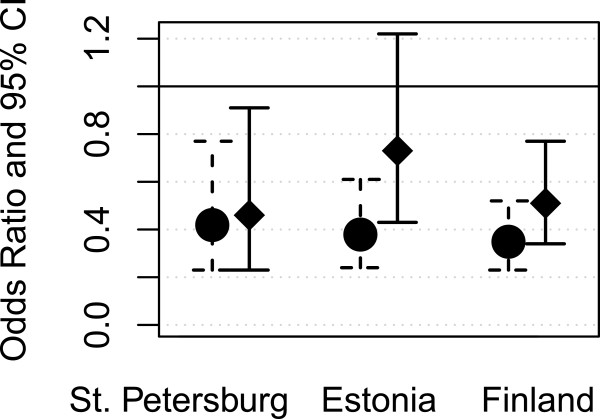
Likelihood (odds ratios and 95% confidence intervals) of poor self-rated health by education (17+ years vs. < 11 years) in the three areas; age-adjusted model on the left and fully adjusted model on the right.

In the age-adjusted models we found a negative association between high education and poor SRH in all studied populations (COR = 0.42 for St. Petersburg women, COR = 0.38 for Estonian women and COR = 0.35 for Finnish women, Figure 1 and Tables 3, 4 and 5). Adjustment for age did not change the association between education and poor SRH, but adjustment for all factors removed the statistical significance in Estonia (Figure 1).

**Figure 2:**
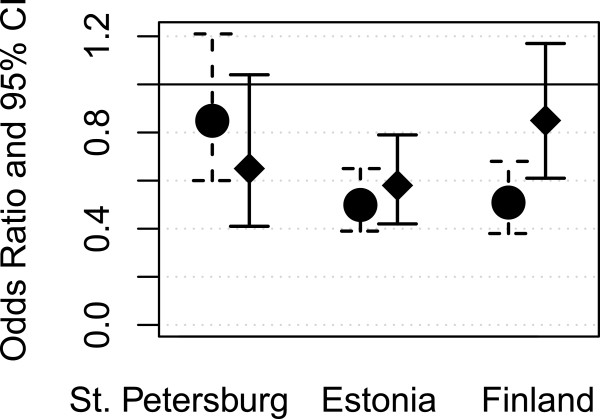
Likelihood (odds ratios and 95% confidence intervals) of poor self-rated health by personal income (highest vs. lowest quartile) in the three areas; age-adjusted model on the left and fully adjusted model on the right.

Adjusting for age, personal income and SRHs did not correlate strongly in St. Petersburg in contrast to Estonia and Finland, Figure 2. Adjustment for all factors (Model IV) removed the statistical significance of income in Finland, whereas in Estonia the significance remained. The interaction between country and personal income was significant (data not shown).

**Figure 3:**
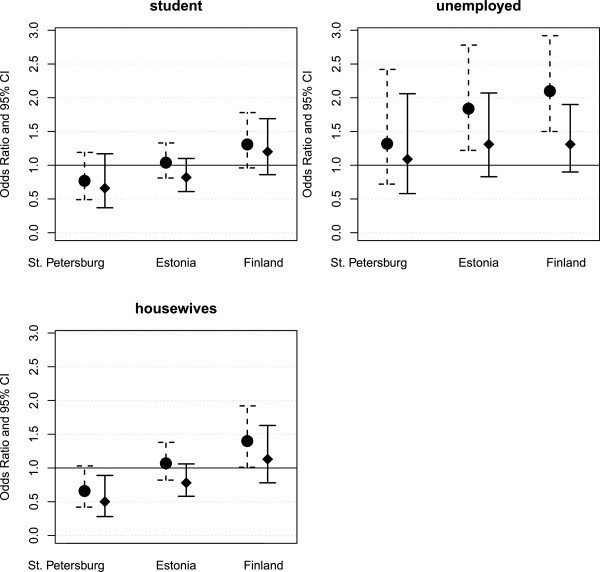
Likelihood (odds ratios and 95% confidence intervals) of poor self-rated health by unemployment (vs. employed) and being a housewife (vs. employed) in the three areas; age-adjusted model on the left and fully adjusted model on the right.

Compared to employed women, unemployment was associated with poor SRH in Estonia and Finland, but not in St. Petersburg, Figure 3. After adjusting for all background characteristics the statistical significance of the association in Estonia and Finland vanished.

In St. Petersburg unlike the other two areas, housewives rather than employed women had less often poor SRH (COR = 0.66). The better SRH of housewives in St. Petersburg remained in the fully adjusted model (Figure 3). The interaction between country and employment status was not significant (data not shown).

Mutual adjustment for the socioeconomic position variables revealed marginally significant interaction between country and employment status, with the interaction between country and income remaining significant and the interaction between country and education remaining insignificant (data not shown).

### Health behaviour and differences in socioeconomic self-rated health

The expected associations between health behaviour factors (BMI and smoking) and poor self-rated health was found in all studied populations (age-adjusted models in Tables 3, 4 and 5). Current smokers had poorer self-rated health in comparison to never smokers. The overweight and obese respondents were more likely to report poorer health in comparison to those with normal weight.

Adjustment for smoking and BMI (Model III vs. Model I) reduced the negative association between education (17+ years vs. < 11 years) and poorer health by 7%, 44% and 18% among St. Petersburg, Estonian and Finnish women, respectively; however, the association between education and health remained significant. Adjustment for health behaviour did not affect the interaction between income and country, although it reduced the significance of the interaction between employment status and country (data not shown).

### Longstanding illnesses and differences in socioeconomic self-rated health

A positive association was found between longstanding illness and poorer self-rated health (age-adjusted models in Tables 3, 4 and 5). Adjustment for chronic limiting conditions reduced the educational health gradient further (Model III vs. Model IV) by 23% for highest (17+ years) vs. lowest (<11 years) education among Estonians, and among Finns by 8%, although among Russians the association remained unchanged. The income health gradient was reduced in Finns and Estonians. Also the incorporation of chronic diseases in the explanatory model reduced the association between unemployment and poorer SRH among Finns, and the association lost its significance.

The interaction between income and country remained significant, p < 0.05, but the modification effect of country on the association between education/employment status and health remained insignificant (data not shown).

## Discussion

In all the studied populations education was associated with SRH; this finding is in accordance with previously reported results from surveys conducted in the former Soviet Union and also in other European countries [34,35]. However, in respect to material economic factors, we found neither a mediating effect of income or employment on the association between education and health nor an independent income–health gradient in St. Petersburg comparable to that found in Finnish and Estonian women. The finding is consistent with the previously reported lack of an explanatory effect of income on the association between education and mortality in Russian women and a relatively weak correlation between income and mortality [36]. It was emphasized that in the Russian settings in the 1990s, inflation, bartering, living on savings, along with a low economic return for education may have distorted the link between income and health that has been consistently reported in other industrialized countries [36]. Our findings are in line with those statements. For that reason, education rather than income could be considered as a meaningful dimension of women’s socioeconomic position in St. Petersburg.

We did not examine the influence of household income on women’s health, which could act as a buffer for the financial difficulties of those with low individual income. This was partly due to anticipated difficulty in interpreting family income, as the concept of family varies between the study areas. In St. Petersburg extended families as economic units were still common and in Finland common law marriages were very common; also taxation is separate for the woman and her partner.

In St. Petersburg housewives rather than employed women had better perceived health, unlike in the two other areas. We do not know what this finding reflects, but it could relate to selection to become a housewife by area.

In all three studied populations the association between socioeconomic position and health was partially explained by smoking habits and relative body weight. This finding suggests that in St. Petersburg lifestyle factors could be mediating factors in the association between social status and perceived health in the same way as in Estonia and Finland. However, it is worth noticing that a selection process could also be involved. In Finland it has been shown that smoking in adolescence predicts poorer educational achievements and affects future socioeconomic status [37]. Thus smoking may act as a factor of indirect selection for the association between education and health and not only mediate, but confound the association between education and health. Psychosocial stress, resulting from disadvantaged socioeconomic position, may affect risk-taking behaviour such as smoking and at the same time cause poor health. Due to this mechanism the contribution of the behavioural factors to the educational health divide may be overestimated.

Limiting longstanding illness may affect the association between socioeconomic position and health via mediating and selecting mechanisms. Unlike in Estonia and Finland, adjustment for longstanding illness did not affect the educational health gradient among women from St. Petersburg. A lower rate of health care utilisation in the lower educational categories [38] may also result in misreporting of longstanding illness by those with poorer education.

Our findings do not allow for a direct formulation of appropriate interventions targeting lower educated women for lifestyle modification so as reduce the educational health divide. However, they show that there may be a need to tailor intervention designs for different countries and areas. Future research regarding such interventions and identification of the potential barriers to healthy choices among women with low socioeconomic position are needed.

### Study strengths and limitations

Strengths of this comparative study include that all surveys were population based and done in close-by years. In Finland the survey was made three years earlier than in the other countries. But there were no major economic or social changes between 2001 and 2004. Surveys were carried out in the context of larger surveys that did not focus on socioeconomic differences, which may reduce any potential reporting bias by the studied question. The questions used were very similar across the studies. The Finnish survey was used as a model and the Estonian and Russian questionnaires were each compiled by the research team with the aim of achieving good comparability, regardless of the different language structures. Furthermore, local researchers were strongly involved in negotiating and arriving at the appropriate word meanings.

The study limitations are those typical of questionnaires and self-reporting: the cross-sectional study design cannot provide evidence on causality; the effect of selection bias cannot be estimated; and presumably a lower participation rate for marginalized groups may lead to underestimation of the contribution of lifestyle factors to health disparities. Furthermore, the response rates were different in the study countries and this may weaken comparisons across countries.

Even though the question on self-rated health was the same in all study areas, it is possible that the meaning of the question and the answer options varied by country due to language and cultural differences. In Finland, data of body weight and height were measured and data of income obtained from tax records. In St. Petersburg and Estonia they were asked from the respondents, and the answers may be less accurate than in Finland. Getting data from tax offices was not possible in St. Petersburg and Estonia, and data collection method did not allow direct measurements.

## Abbreviations

BMI: Body mass index; OR: Odds ratio; SRH: Self–rated health; COR: Cumulative odds ratio.

## Competing interests

The authors declare that they have no competing interests.

## Authors’ contributions

TD participated in the data collection (St. Petersburg), originated the idea, planned the analysis and prepared the draft manuscript. TH carried out the analysis and commented the manuscript. ER participated in the data collection (St. Petersburg), finalized the article and commented the manuscript. EH participated in designing the study and commented the manuscript. EH-M participated in designing the study and commented the manuscript. ML participated in the data collection (Estonia) and commented the manuscript. OK participated in designing the study and commented the manuscript. SK participated in the data collection (Finland), designing the study and commented the manuscript. All authors read and approved the final manuscript.
